# Quality Appraisal of Telerehabilitation Guidelines: A Systematic Review

**DOI:** 10.1155/ijta/2404971

**Published:** 2026-06-24

**Authors:** Faridokht Salahshoori, Majid Jangi, Ebrahim Sadeghi-demneh, Farhad Fatehi, Alireza Rahimi

**Affiliations:** ^1^ Medical Library and Information Science Department, Health Information Technology Research Center, Isfahan University of Medical Sciences, Isfahan, Iran, mui.ac.ir; ^2^ Health Information Technology Research Center, Isfahan University of Medical Sciences, Isfahan, Iran, mui.ac.ir; ^3^ Musculoskeletal Research Center, Isfahan University of Medical Sciences, Isfahan, Iran, mui.ac.ir; ^4^ Business School, The University of Queensland, Brisbane, Australia, uq.edu.au

**Keywords:** guideline, quality app, telerehabilitation

## Abstract

**Background:**

Telerehabilitation is an expanding domain of telehealth offering remote rehabilitation services. The quality of clinical guidelines, despite significant growth, is highly inconsistent, which may affect the quality of care and policy execution. We endeavored to systematically assess the quality of general telerehabilitation guidelines using the AGREE II instrument.

**Methods:**

A systematic search of six databases and grey literature identified 2575 records. After screening and eligibility assessment, seven guidelines were appraised independently by two reviewers using the AGREE II tool, covering six quality domains.

**Results:**

Only the American Physical Therapy Association′s 2024 guideline was rated high quality across all domains. Three guidelines were rated medium quality and recommended with modifications. The remaining three were deemed low quality due to weak methodological rigor and limited stakeholder involvement.

**Conclusion:**

The quality of telerehabilitation guidelines differs significantly, and high‐quality, evidence‐based, and inclusive guidelines are essential for promoting secure, efficient, and telerehabilitation practices globally.

## 1. Introduction

Telerehabilitation, a branch of telehealth, employs digital technologies to remotely provide rehabilitation services [1]. This methodology grew considerably, especially following worldwide challenges, such as the coronavirus disease (COVID‐19) epidemic, which disrupted conventional in‐person care [2]. Telerehabilitation offers the potential to enhance access to rehabilitation interventions, improve continuity of care, and decrease healthcare costs for patients in remote or underserved regions [3]. However, the success of its performance depends on comprehensive standards that ensure its safety, effectiveness, and equitable distribution. Despite the growing deployment of telerehabilitation, concerns remain regarding the standardization and quality of telerehabilitation guidelines that regulate its application. Furthermore, the quality of telerehabilitation guidelines affects patient outcomes, professional confidence, and telehealth policy development [4, 5].

High‐quality guidelines are essential for standardizing telerehabilitation practices to ensure that they are evidence‐based, patient‐centered, and aligned with professional standards [6]. Without clear, well‐developed guidelines, telerehabilitation implementation risks variability, which may compromise care quality and safety [4]. Guidelines are critical tools for training healthcare providers, fostering interdisciplinary collaboration such as clinical informationists [7, 8] and guiding the integration of innovative technologies into rehabilitation practices [9–12]. Ensuring the quality of telerehabilitation guidelines and improving their reliability is essential for building trust among healthcare professionals and patients, ultimately facilitating a greater global adoption of telerehabilitation [6, 13].

Although the potential of telerehabilitation to transform healthcare delivery is widely acknowledged, the quality of the guidelines that underpin this approach has received little attention [14–17]. Existing research has primarily focused on the technological, clinical, and operational aspects of telerehabilitation but has largely overlooked a systematic evaluation of the guidelines′ rigor and methodological soundness [18]. To the best of our knowledge, no prior review has assessed quality of general telerehabilitation guidelines and contributes a new perspective to the discourse on telerehabilitation [15]. This deficiency underscores the necessity for a thorough appraisal to elucidate the current status of these guidelines and to identify areas requiring improvement.

The current research is aimed at addressing a significant gap in the literature by systematically assessing the quality of general telerehabilitation guidelines using the Appraisal of Guidelines for Research and Evaluation II (AGREE II) instrument. The review and evaluation of current telerehabilitation guidelines identify unmet rehabilitation needs that telerehabilitation could address. This study informs the development of enhanced recommendations by constructively critiquing the guidelines, weighing their advantages and disadvantages, and providing evidence‐based suggestions for improvement, which ultimately aims to enhance telerehabilitation quality and safety for policymakers, clinicians, clinical informationists, and stakeholders. This, in turn, promotes the practical application of telerehabilitation guidelines by ensuring adherence to high‐quality standards, improving patient outcomes, and fostering innovation. Specifically, this study systematically assesses the quality of general telerehabilitation guidelines using the AGREE II instrument, addressing a significant gap in the literature. This study will promote the practical application of telerehabilitation guidelines by guaranteeing adherence to high‐quality standards, improving patient outcomes, and fostering healthcare delivery innovation.

## 2. Method

### 2.1. Search Strategy

A comprehensive search was conducted in six electronic databases: PubMed, Scopus, ISI, Embase, ProQuest, and Cochrane. The electronic search strategy was developed and conducted in collaboration with an experienced database specialist (see Data S1 for the full search strategy). The electronic search was conducted in English language and with time limits from 2002 to 2024. Additional research was conducted on Google for national and organizational government telerehabilitation guidelines. Reference lists of included guidelines were checked to identify additional documents relevant to telerehabilitation guidelines. The search strategy is presented in Supporting Information section.

### 2.2. Inclusion and Exclusion Criteria

This study included guidelines published in English between 2002 and 2024 and included only general guidelines for telerehabilitation. In this review, “general telerehabilitation guidelines” were described as overarching documents providing recommendations applicable across various rehabilitation settings, encompassing governance, ethical principles, technical infrastructure, service delivery models, professional responsibilities, and implementation considerations, rather than focusing on guidelines specific to a particular diagnosis. We excluded guidelines that focused on specific patients and telemedicine guidelines, as well as any documents that were not guidelines.

### 2.3. Data Extraction

A total of 2575 records were identified through database searches, with an additional 10 through other sources. Following the removal of duplicates (1053) in EndNote 21, the remaining studies were divided into two groups (included and excluded) by using the Smart Groups function in EndNote. One thousand, five hundred and twenty‐two studies were identified and screened title or abstract. One thousand, four hundred and eight studies were excluded because they did not meet the inclusion criteria. Abstract screening was performed by two authors independently, and 41 full‐text studies were inspected by authors for eligibility. Finally, seven telerehabilitation guidelines were included in the study.

### 2.4. Data Analysis

AGREE II instrument is an internationally validated and widely adopted tool designed to assess the methodological quality and transparency of clinical practice guidelines. AGREE II evaluates guidelines across six domains: “scope and purpose,” “stakeholder involvement,” “rigor of development,” “clarity of presentation,” “applicability,” and “editorial independence.” These domains collectively examine both the process of guideline development and the quality of reporting, ensuring that recommendations are evidence‐based, transparent, and implementable. The instrument has been extensively used across diverse healthcare fields, including telehealth and rehabilitation, and is considered the gold standard for guideline quality appraisal [19]. Two assessors (one expert in the telerehabilitation system and one expert in the development of guidelines) independently evaluated the included guidelines using the AGREE II instrument, which consists of 23 items in six domains. Each item was rated on a seven‐point scale from 1 (*strongly disagree*) to 7 (*strongly agree*). The domain score is calculated by summing all the scores of the individual items in a domain and by scaling the total as a percentage of the maximum possible score for that domain. The standardized domain percentage was then computed using the following formula: standardized  domain  score  (*%*)  =  (obtained  score   −  minimum possible score)/(maximum  possible  score  −  minimum possible score) × 10.0. The same procedure is performed for the total overall score, using the total domain scores for the computation. The mean scores of AGREE II instrument items for each guideline are shown in Supporting Information section, Table S1. Discrepancies in scoring were discussed between the reviewers, and consensus was achieved through deliberation. In cases where disagreement persisted, a third senior reviewer was consulted to reach a final decision. Although the formal interrater reliability statistics were not calculated, the independent assessment and consensus process were applied to enhance the rigor and transparency of the appraisal. On completing the 23 items, the appraisers provided the overall assessment of each guideline, and decided which guideline was recommendable, with or without modifications, and which was not recommendable.

No set thresholds exist for determining high‐/low‐quality guidelines; however, AGREE II guidance suggests users decide these according to their specific context. The domain score is calculated by summing all the scores of the individual items in a domain and by scaling the total as a percentage of the maximum possible score for that domain. The same procedure is performed for the total overall score, using the total domain scores for the computation [20]. Based on the examples given in the AGREE II user manual [21] and with reference to previous studies [22, 23], we considered domain and overall scores under 40% to indicate poor quality, 40%–59% medium quality, 60%–80% good quality, and above 80% excellent.

Finally, a qualitative content analysis was conducted to synthesize recommendations across the telerehabilitation guidelines included in the study. The relevant recommendations were extracted verbatim or near‐verbatim from each guideline and organized into a comparison matrix. Recommendations addressing similar concepts were grouped into common categories, whereas divergent or inconsistent recommendations were classified as discordant. In addition to extracting explicit recommendations, the qualitative synthesis was structured using a subsystem‐based framework. In this context, “subsystem‐based” refers to organizing the extracted recommendations into predefined functional domains of telerehabilitation systems (e.g., governance, clinical delivery, technical infrastructure, and professional roles). This structuring approach was used solely to enhance conceptual clarity and comparability across guidelines and does not represent an inductive thematic analysis.

This process did not involve inductive coding or formal thematic analysis; rather, it is aimed at providing a transparent descriptive synthesis of guideline content. All categorizations were discussed and agreed upon by the review team to ensure consistency and accuracy.

## 3. Results

### 3.1. Guideline Selection

**Figure 1:**
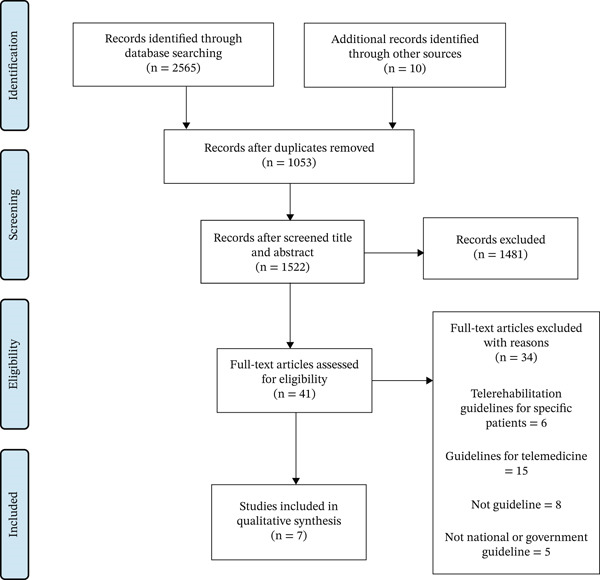
Preferred Reporting Items for Systematic Reviews and Meta‐Analyses (PRISMA) flowchart.

Figure 1 displays the PRISMA diagram illustrating the process of identifying, screening, and selecting the included studies. In total, 2575 articles were identified from the main databases and other grey literature sources. After removing 1053 duplicates, 1522 articles were eligible for screening. Following the eligibility criteria, 1481 articles were excluded after the title and abstract evaluation. After excluding these articles, 41 articles were read in full to determine eligibility, and 31 records were removed as irrelevant. Then, the full texts of 41 articles were reviewed for eligibility, resulting in the exclusion of 34 records deemed irrelevant. Ultimately, seven relevant guidelines containing recommendations for rehabilitation services were included in the final analysis [5, 24–28].

### 3.2. Guideline Characteristics

The systematic search identified seven guidelines that met the inclusion criteria. Table 1 summarizes the key characteristics of the included telerehabilitation guidelines, including publication year, organizational context, target audience, scope, and methodological approach.

**Table 1:** The key characteristics of the included telerehabilitation guidelines.

Guideline	Country	Year of publication	Target audience	Scope	Development method
A Blueprint for Telerehabilitation Guidelines	The United States	2010	Professional providers of telerehabilitation services	Administrative, clinical, technical, and ethical principles of telerehabilitation services	Expert consensus
American Telemedicine Association′s Principles for Delivering Telerehabilitation Services	The United States	2017	Professional providers of telerehabilitation services	Administrative, clinical, technical, and ethical principles of telerehabilitation services	Expert consensus
Telehealth: Clinical Guidelines and Technical Standards for Telerehabilitation	Canada	2006	Professional providers of telerehabilitation services	Clinical and technical standards for telerehabilitation services	Interdisciplinary rehabilitation team panel
Telerehabilitation Guidelines in Saudi Arabia	Saudi Arabia	2021	Professional providers of telerehabilitation services	Administrative, clinical, technical, and ethical principles of telerehabilitation services	Expert consensus
Modalities for the Implementation of Telerehabilitation	Luxembourg	2022	Professional providers of telerehabilitation services	General principles (clinical, environmental/human, technological, and organizational factors)	Expert consensus
Telerehabilitation Guide	Canada	2024	Physiotherapist and providers of telerehabilitation services	Regulatory requirements, legislation, and standards of telerehabilitation services	Expert consensus
Telerehabilitation in Physical Therapist Practice: A Clinical Practice Guideline from the American Physical Therapy Association	The United States	2024	Physiotherapist and providers of telerehabilitation services	International clinical practice guideline of telerehabilitation	Systematic review of published studies

Seven telerehabilitation guidelines were reviewed from diverse countries, including the United States, Canada, Luxembourg, and Saudi Arabia. The guidelines were developed by national and international professional organizations (e.g., American Physical Therapy Association [APTA] and American Telemedicine Association [ATA]), ministries of health (e.g., Ministry of Health, Saudi Arabia), regulatory authorities (e.g., College of Physical Therapist of British Columbia [CPTBC]), and international nongovernmental organizations (e.g., Humanity & Inclusion), demonstrating a global, interdisciplinary approach to telerehabilitation services.

Although the aims and scope of these guidelines were quite closely aligned around offering safe, effective, and ethical telerehabilitation services, their development methods differed widely, from systematic reviews (e.g., APTA) to formal articles (e.g., ATA and Blueprint) to institutional consensus documents (e.g., Saudi national guideline). Furthermore, some of the guidelines, such as the APTA and Canadian CPTBC guidelines, were specific to physiotherapy, whereas others were interdisciplinary and could be utilized by a wider range of rehabilitation professionals, including occupational and speech therapists.

Despite methodological differences in the included guidelines, their content was aligned in several common areas, including clinical practice standards, administrative protocols, technical infrastructure, ethical considerations, and compliance with legal requirements. The guidelines′ administrative content tended to address recommendations in terms of medical record management, informed consent procedures, standards of supervision, and interprofessional collaboration. The clinical aspects focused on issues such as patient assessment, intervention planning, follow‐up care, and various fully remote or hybrid service delivery models.

Most guidelines have broadly focused on telerehabilitation without targeting specific diagnostic groups. Several guidelines also highlighted the importance of flexible service delivery models, including fully remote and hybrid approaches, and emphasized the relevance of community‐based models of care. Additionally, guidelines developed in resource‐limited contexts or with a humanitarian perspective (such as the HI and Saudi Arabia documents) have provided important insights about digital access constraints and the importance of using a community‐based service delivery model.

Only a limited number of guidelines have reported the use of systematic evidence synthesis regarding methodological foundations in their development process. For example, the APTA 2024 guideline adopted a systematic approach to evidence review, whereas most other documents relied primarily on expert consensus, regulatory frameworks, or organizational policy statements. Similarly, the CPTBC guideline did not report a formal evidence synthesis process but provided detailed guidance on the regulatory, technical, and clinical components of remote rehabilitation practice.

Although the majority of documents had a strong focus on foundational principles such as client‐centered care, adherence to safety and privacy, and ongoing professional development of healthcare, there were distinctions in levels of clinical expertise or utilization of scientific evidence and implementation items across guidelines. Among these differences, only the APTA guideline provided specific clinical recommendations based on clinical conditions supported by valid, peer‐reviewed research evidence, whereas the other guidelines provided a policy framework or general guidelines about optimal practice management.

### 3.3. Quality Assessment of Included Guidelines

**Figure 2:**
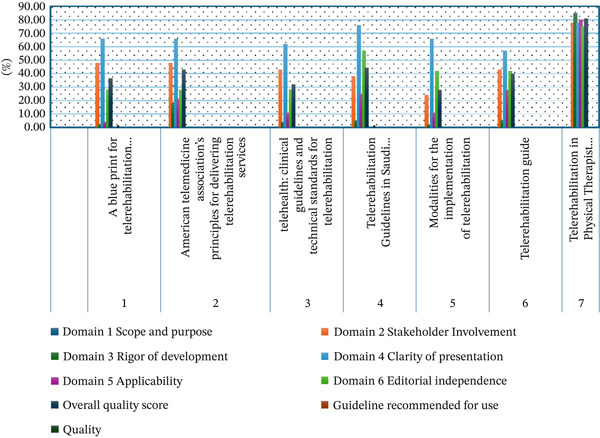
Scores for each domain of guidelines by the Agree II tool.

The AGREE II instrument was used to evaluate the methodological quality of the included guidelines across six domains: scope and purpose, stakeholder involvement, rigor of development, clarity of presentation, applicability, and editorial independence. Table 2 and Figure 2 present the AGREE II domain scores for each guideline and their overall quality classification based on domain scores.

**Table 2:** Scores for each domain of the guidelines by the Agree II tool.

Guideline name		Domain 1: Scope and purpose	Domain 2: Stakeholder involvement	Domain 3: Rigor of development	Domain 4: Clarity of presentation	Domain 5: Applicability	Domain 6: Editorial independence	Overall quality score	Guideline recommended for use	Quality
**1**	**A Blueprint for Telerehabilitation Guidelines**	71%	48%	2%	66%	4%	28%	36.5%	No	Poor
2	**American Telemedicine Association′s principles for delivering telerehabilitation services**	76%	48%	18%	66%	21%	28%	43%	Yes, with modification	Medium
3	**Telehealth: Clinical Guidelines and Technical Standards for Telerehabilitation**	43%	43%	4%	62%	11%	28%	32%	No	Poor
4	**Telerehabilitation Guidelines in Saudi Arabia**	66%	38%	5.3%	76%	25%	57%	44.5%	Yes, with modification	Medium
5	**Modalities for the implementation of telerehabilitation**	24%	24%	2%	66%	11%	%42	28%	No	Poor
6	**Telerehabilitation guide**	66%	43%	5.3%	57%	28%	42%	40%	Yes, with modification	Medium
7	**Telerehabilitation in Physical Therapist Practice: A Clinical Practice Guideline from the American Physical Therapy Association**	90%	78%	85%	78%	80%	75%	81%	Yes	Excellent

Of the seven guidelines evaluated, only one, that is, Telerehabilitation in Physical Therapist Practice (APTA, 2024), achieved a high score across all domains and was rated as high quality overall. This guideline scored > 70% in all six AGREE II domains, 90% scope and purpose, and 85% rigor of development. It was the only guideline to achieve the high‐quality criteria for Domain 3 (rigor of development) and Domain 6 (editorial independence). It also received high scores for Domain 2 (stakeholder involvement) and Domain 5 (applicability) at 78% and 80%, respectively. This guideline demonstrated particularly strong performance in the domains of rigor of development and applicability, reflecting a systematic evidence‐based development process and clearer implementation guidance.

Three guidelines received fair quality scores and are recommended for use with revisions. They showed acceptable performance (≥ 50%) in Domain 1 (scope and purpose) and Domain 4 (clarity of presentation). The following three guidelines were developed: the ATA (2017), the Saudi national guideline (2021), and the CPTBC (2024). Each guideline clearly stated the purpose and presented alignments in the overall structure; however, all three were markedly lower in Domain 2 (stakeholder involvement) and Domain 3 (rigor of development) and demonstrated scores from moderate to low for applicability and editorial independence. Its methodological limitations negatively impacted the overall quality classification and suggest that they will need targeted intervention before widespread implementation. These guidelines generally showed acceptable clarity of presentation but demonstrated weaknesses in stakeholder involvement and evidence synthesis processes, indicating the need for targeted improvements before widespread implementation.

Three guidelines received low‐quality scores and were not recommended for use at this time. An example of this is A Blueprint for Telerehabilitation Guidelines, Telehealth Guidelines by ISfTeH, and Modalities for the Implementation of Telerehabilitation (Humanity & Inclusion). Although they showed some strengths in scope and clarity of presentation, all three failed to meet the requirements for good quality with respect to rigor of development and stakeholder involvement (with scores below 30% in each domain). Specifically, the ISfTeH guideline achieved only 4% development rigor, whereas the Humanity & Inclusion guideline recorded only 2% in both stakeholder engagement and rigor, suggesting a lack of methodological structure and transparency in the guideline development process. Although both the ISfTeH and Humanity & Inclusion guidelines recorded clear clarity scores (e.g., 66%), their overall scores were 36.5%, 32%, and 28%, respectively, due to significant methodology flaws, which affect their reliability and applicability in evidence‐based practice. These guidelines showed limited transparency in their development processes and insufficient consideration of applicability and stakeholder engagement, which may compromise their reliability and relevance in evidence‐based practice.

The AGREE II results indicate that methodological quality is closely related to the guideline development approach and institutional context when interpreted together with the characteristics summarized in Table 1. Guidelines developed using systematic evidence‐based methods tended to achieve higher domain scores, whereas those primarily based on expert consensus or policy‐driven approaches demonstrated lower methodological rigor.

Furthermore, Figure 2 illustrates the distribution of AGREE II domain scores across guidelines, highlighting both strengths and gaps in current telerehabilitation guidance. These findings reveal the heterogeneity of existing guidelines and underscore the important limitations of the methodological rigor, stakeholder involvement, and implementation‐oriented design.

### 3.4. Summary of Recommendations

A qualitative synthesis of recommendations was conducted across the included telerehabilitation guidelines, and specific recommendations for telerehabilitation practice were synthesized from the reviewed guidelines. They include both common messages that are shared across multiple documents and inconsistent messages that differ in approach or emphasis. The synthesized findings are presented below, with explicit references to the source guidelines to ensure transparency and traceability.

#### 3.4.1. Common Messages

Several consistent recommendations for telerehabilitation service delivery are identified across the guidelines that were reviewed:

1. Client‐centered care and ethics: All guidelines emphasize the need to provide individualized care that is based on a person′s needs and grounded in ethics, including being guided by principles of autonomy, beneficence, confidentiality, and informed consent [5, 24–28].
2. Clear guidelines for use of telerehabilitation*: Seven of the documents raise the need for assessing whether telerehabilitation is appropriate on a case-by-case basis, considering the person′s condition, goals, access to technology, and safety* [5, 24–28].
3. Digital infrastructure and data security: All guidelines endorse technologies that are secure, stable, and compliant. They referenced the importance of some aspect of data protection, privacy protections, and regulation [5, 24–28].
4. Professional competency and accountability: Providers must have appropriate clinical and technical skills to provide remotely delivered care. All guidelines emphasize training in digital literacy and adherence to professional standards [5, 24–28].
5. Documentation and billing: All guidelines recommend documentation and billing that is accurate, transparent, and compliant with regulations, consistent with delivering care in‐person [5, 24–28].
6. Interprofessional collaboration: Interprofessional teams should be involved in planning and delivering telerehabilitation, as recommended by many guidelines [5, 24–28].

#### 3.4.2. Discordant Messages

Although there is general agreement in the areas highlighted above, there are differences in the recommendations in various aspects:

1. Involving stakeholders in the development of guidelines: Only a few guidelines involve patients or stakeholders across diverse professions in the development process. This leads to variation in the focus on aspects of cultural relevance, user experience, and inclusion [24–28].
2. Quality of evidence: There are significant differences in methodological quality. Some guidelines (e.g., APTA) provided evidence‐based recommendations from a systematic review, whereas others are based primarily on expert consensus [24–28].
3. Specificity of clinical recommendations: Some guidelines were very prescriptive; thus, the recommendations are for clinical protocols (e.g., assessment tools and management of adverse events), and others provide general guidelines with no actionable steps [5, 26–28].
4. Feasibility in resource‐limited settings: Although several guidelines acknowledge contextual challenges such as limited internet access, digital literacy, and infrastructure constraints, there is substantial variation in the depth and specificity of recommendations addressing these issues. Some documents merely recognize these barriers without providing operational guidance, whereas others propose more concrete mitigation strategies [5, 26–28].

## 4. Discussion

The current systematic review identified, synthesized, and critically appraised seven international clinical practice guidelines on telerehabilitation. The guidelines were created between 2006 and 2024 in a diverse range of contexts (country and institution) to support the global and interdisciplinary aspects of telerehabilitation. These guidelines were produced by national and international professional organizations (e.g., APTA and ATA), regulatory agencies (e.g., CPTBC), health ministries (e.g., Saudi Arabia), and international nongovernmental organizations (e.g., Humanity & Inclusion) with respect to practice contexts and audiences. Based on their methodological quality and content analysis, we extracted primary recommendations for practice and identified evidence of consensus across the documents. This synthesis offers clinicians, health systems planners, professional associations, and policy makers a consolidated picture of current standards and expectations for telerehabilitation practice around the world.

Although the overarching aims of the guidelines were to ensure the safe, effective, and ethically sound use of telerehabilitation services, there was significant heterogeneity between methodological approaches and structural aspects of the guidelines. For example, only the APTA guideline developed (APTA, 2024) used a systematic review of over 5000 documents to inform the guideline and had the level of methodological rigor. The other guidelines were mostly driven by expert consensus or positional statements of the organization; many did not adequately describe the evidence synthesis process and did not demonstrate stakeholder involvement in the development process.

Consistent with our findings, Anil et al. [29] in their rapid scoping review identified significant gaps in guidance around telerehabilitation, particularly for movement‐related assessments of people with physical disabilities. Among the 23 documents they reviewed, few provided recommendations specific to the age or condition of the participant, and did not provide any detail at the subsystem level about the types of training for providers, the technological aspects, or the support to clients. Implementation considerations were almost nonexistent, and movement‐specific assessment training was consistently minimal. The use of synchronous delivery modes and variation in session structure demonstrates a lack of standardization. In this study, the modular, subsystem‐based framework is applied as an analytical approach that decomposes telerehabilitation guidelines into interconnected functional subsystems (e.g., client‐centered care and ethics, case‐by‐case appropriateness assessment, digital infrastructure and data security, professional competency and accountability, documentation and billing, and interprofessional collaboration) to systematically identify strengths and gaps across guidelines. Overall, these findings support the continued relevance of this framework as a pragmatic analytical model for evaluating and improving telerehabilitation guidelines [29].

Our findings are also consistent with the results of the systematic review completed by Appleby et al. regarding the impact of telerehabilitation of adults after stroke. The review found that 13 randomized controlled trials demonstrated a generally positive effect on motor function, activities of daily living, and satisfaction, with the majority of experimental groups demonstrating a positive impact. However, both reviews noted considerable heterogeneity in terms of the type of interventions, mode of delivery, structure of sessions, and outcome measures. Although videoconferencing was the primary mode or method of service delivery, there is a variation in the absence of a standardized intervention framework and the variability or lack of information regarding provider training, technology infrastructure, and support to improve replicability or scalability to replace current telerehabilitation models. Also, both reviews identified methodological limitations, including undefined or unjustified sample size, risk of cointervention, and limited involvement of caregivers. In summary, the literature provides evidence that demonstrates that a standardized, system‐based design, like we proposed, is needed to capture and evaluate the efficacy, reparability, and scalability of telerehabilitation programs, which is even more demanding for more complex populations, such as those in pediatric populations or those with cognitive impairment. Notably, although Appleby et al. [30] used intervention studies in order to rate their evidence, we utilized and systematically organized and synthesized clinical practice guidelines to assess the coverage, structure, and support of current implementation.

The convergence of important themes in the guidelines reviewed, such as client‐centered care, ethical practice, digital security, and provider competence, demonstrates an underlying agreement in telerehabilitation practice. These consistent messages are congruent with national and global standards established by the World Health Organization (WHO) (2019) and the ATA (2020), which endorse ethical, secure, competent digital health delivery [31, 32]. The emphasis on individualized care, informed consent, and privacy reflects a shared commitment to respect patient autonomy and welfare remotely [31, 32].

Likewise, the focus on strong digital infrastructure and data security is consistent with findings from studies such as Kruse et al. [33], which identified the need for privacy and technical dependability as key barriers to telehealth use. The recurrent theme of provider training and accountability is also supported by evidence from Greenhalgh et al. [34], who emphasized that clinician readiness is a fundamental factor of integrating telehealth. Nevertheless, most guidelines do not critically assess whether their technical requirements align with international standards or global recommendations, such as those of the WHO. Many specifications implicitly reflect high‐resource settings with advanced digital infrastructure, limiting their applicability across diverse contexts. The feasibility of these technical expectations in low‐resource and underserved settings is rarely addressed. This lack of contextual adaptation may restrict the scalability and equitable implementation of telerehabilitation services. These findings underscore the importance of developing context‐sensitive, resource‐adaptive technical guidance that supports equitable implementation across diverse health systems. Moreover, although several guidelines recognize the relevance of telerehabilitation in resource‐limited settings, few explicitly examine how contextual factors affect their applicability. Variations in digital infrastructure, device availability, internet stability, and digital literacy among providers and service users can substantially influence the feasibility of guideline implementation. Insufficient attention to these contextual determinants may limit guideline transferability and contribute to inequities in access to telerehabilitation services.

However, inconsistencies in stakeholder participation, evidentiary adequacy, recommendation levels, and context specificity reveal significant gaps in the guidelines. The limited role of patients or caregivers in guideline development raises concerns about cultural appropriateness, accessibility, and user experience. Several factors may explain the limited stakeholder involvement in guideline development, including reliance on expert‐driven processes, time and resource constraints, and the rapid evolution of telerehabilitation practices. The exclusion of patients, caregivers, and diverse rehabilitation professionals may reduce guideline usability, contextual relevance, and real‐world applicability. Consequently, insufficient stakeholder engagement can hinder guideline adoption and implementation, particularly in complex or underserved settings. These issues were emphasized by multiple studies that promote or recommend participatory processes in digital health policy development [35–37]. Also, the inconsistency in the methodological rigor of guidelines, with only a few using systematic evidence synthesis (e.g., APTA), is a concern regarding the quality of clinical practice guidelines. The ability in clinical specificity of the guidelines, ranging from detailed protocols to conceptual guidance, reduces the usability of guidelines for frontline practitioners. This reflects the results of Papadopoulos et al. [38], who highlight operational specificity as an essential characteristic for the successful translation of digital health policies into practice.

These issues justify our modular, subsystem‐based analysis, which considered content, structure, and development. This approach highlighted specific areas where current telerehabilitation frameworks inadequately address implementation complexities and identified opportunities for improvement, particularly regarding methodological rigor, stakeholder engagement, contextual adaptation, and clinical implications.

## 5. Strengths and Limitations

This systematic review makes a new contribution to the digital health literature by looking specifically at clinical practice guidelines for telerehabilitation, rather than reviewing individual intervention studies. In focusing on clinical practice guidelines, the review provided relevant insights into the governance, structure, and implementation frameworks of what real‐world practice looks like. By using a subsystem‐based analytical framework, it offered a more nuanced exploration of under‐explored aspects such as information transfer, monitoring and control, and support services; none of which are likely to be examined as part of a wider evaluation. In addition to providing clinical practice guidelines from both national and international perspectives, the review enabled findings to be more generalizable to different health systems. Using standardized quality appraisal tools (e.g., AGREE II) meant methodological quality could be appraised rigorously and transparently.

There are some limitations to consider. First, this review was subject to language and publication bias, as only English‐language documents were included. Second, the differences in guideline scope, presentation, and level of detail presented challenges in doing any direct comparisons. Although this review focused on “general” telerehabilitation guidelines, some documents were developed for specific rehabilitation professions (e.g., physiotherapy). These guidelines addressed the cross‐cutting and transferable aspects of telerehabilitation practice, but their focus on professional perspectives may restrict the full applicability of certain recommendations across all rehabilitation fields. In addition, nearly all of the guidelines did not reflect on their development process, or stakeholders were involved, which casts doubt on our ability to appraise the contextual validity. An additional limitation of this review is the lack of evidence regarding the real‐world implementation and adoption of the included guidelines. Although this study focused on the methodological quality and content of telerehabilitation clinical practice guidelines, most of the reviewed documents did not report whether their recommendations had been formally evaluated, implemented, or adopted within health systems. Consequently, the extent to which higher‐quality guidelines translate into effective, scalable, or sustainable telerehabilitation practice remains unclear. Lastly, although this review presents structural and content gaps, it did not measure any guidelines implementation in a real‐world context or look at the effectiveness of the guidelines, which will be an area for further research.

## 6. Conclusion

The systematic review demonstrates that the global interest in developing standards for the delivery of telerehabilitation services that are safe, effective, and ethical is increasing. Although general agreement exists on foundational principles, including patient‐centered care, digital infrastructure, provider competence, and data security, there is substantial variability in the methodological rigor, evidence‐base, and clinical specificity present in the guidelines that exist. The review highlights a clear need for the development of high‐quality, inclusive, and relevant telerehabilitation guidelines. Future guideline development should ensure methodological transparency, involve multidisciplinary stakeholders, and adaptation to vulnerable populations and limited‐resource settings occur. To support safe, equitable, and scalable telerehabilitation globally, future guidelines must be high quality, inclusive, and rigorously developed.

## Funding

This study was supported by the Isfahan University of Medical Sciences.

## Disclosure

Dr. Alireza Rahimi affirms that this manuscript is an honest, accurate, and transparent account of the study being reported; that no important aspects of the study have been omitted; and that any discrepancies from the study as planned (and, if relevant, registered) have been explained.

## Ethics Statement

Approval was granted by the Scientific Committee of Isfahan University of Medical Sciences, with the research ethic code IR.MUI.NUREMA.REC.1401.166,

## Conflicts of Interest

The authors declare no conflicts of interest.

## Supporting information


**Supporting Information** Additional supporting information can be found online in the Supporting Information section. Supporting Information. Supporting Information includes two data: Data S1: Search strategy provides the detailed search strings used for the systematic literature review, and Data S2: Table S1 presents the mean scores of the 23 items of the AGREE II instrument for each guideline. This table supports the quality appraisal results summarized in the main text.

## Data Availability

The data that support the findings of this study are available on request from the corresponding author. The data are not publicly available due to privacy or ethical restrictions.
