# Supplementary material for: Quality Appraisal of Telerehabilitation Guidelines: A Systematic Review
Source: Int J Telemed Appl. 2026 Jun 24;2026:2404971. doi: 10.1155/ijta/2404971 (PMC13292017; doi:10.1155/ijta/2404971)
Supplement: Supplementary file 1 — Supporting Information Additional supporting information can be found online in the Supporting Information section. Supporting Information. Supporting Information includes two data: Data S1: Search strategy provides the detailed search strings used for the systematic literature review, and Data S2: Table S1 presents the mean scores of the 23 items of the AGREE II instrument for each guideline. This table supports the quality appraisal results summarized in the main text. [file IJTA-2026-2404971-s001.docx]

**Appendix A: Search Strategy**

("telerehabilitation*" OR "Tele-rehabilitation" OR "Tele rehabilitation*" OR "Remote Rehabilitation*" OR "Virtual Rehabilitation*" OR "Teleconsult*" OR "Tele-consult*" OR "E-consultat*" OR "Remote Consultation*" OR "Remote Rehabilitation*" OR "Virtual Rehabilitation*" OR "Tele-speech" OR "Tele-speech" OR "telerehab" OR "tele-rehab" OR telemedicine OR " Mobile Health" OR mhealth OR "m-Health" OR cellphone OR phone OR "Tele-health" OR telehealth OR ehealth OR "e-Health" OR telemonitor* OR "Tele monitor*" OR "Tele-monitor*" OR "Tele homecare" OR "Tele-homecare" OR telehomcare OR in-home OR (home AND base*) OR home-base* OR telecare OR "Tele-care" OR "Telecommunication*" OR "Distance Counsel*" OR "Distance E Therapy" OR "E -Therap*" OR "E-Counseling" OR "E Counseling" OR "Remote Sensing Technolog*") AND ( guideline* OR standard* OR protocol* )

**Appendix B:**

**Table B1. Mean Scores of AGREE II Instrument Items for Each Guideline**

| **Domain** | **Items of**  **AGREE II** | **Mean Scores of AGREE II Item-Level for Included Guidelines** | | | | | | |
| --- | --- | --- | --- | --- | --- | --- | --- | --- |
|  |  | **A blueprint for telerehabilitation guidelines score** | **American Telemedicine Association’s principles for delivering telerehabilitation services score** | **telehealth: clinical guidelines and technical standards for telerehabilitation**  **score** | **Telerehabilitation Guidelines in Saudi Arabia score** | **Modalities for the implementation**  **of telerehabilitation score** | **Telerehabilitation guide score** | **Telerehabilitation in Physical Therapist Practice: A Clinical Practice Guideline from the American Physical Therapy Association score** |
| **1. Scope and purpose** | **1. The overall objective(s) of the guideline is (are) specifically described** | **7** | **7** | **6** | **7** | **3** | **6** | **7** |
|  | **2. the health question(s) covered by the guideline is (are) specifically described** | **5** | **6** | **3** | **4** | **2** | **5** | **6** |
|  | **3. The population (patients, public, etc.) to whom the guideline is meant to apply is specifically described.** | **6** | **6** | **3** | **6** | **3** | **6** | **7** |
| **2.Stakeholder**  **Involvement** | **4. 4. The guideline development group includes individuals from all relevant professional groups** | **5** | **5** | **5** | **3** | **4** | **4** | **6** |
|  | **5. The views and preferences of the target population (patients, public, etc.) have been sought.** | **2** | **2** | **2** | **2** | **2** | **2** | **6** |
|  | **6. The target users of the guideline are clearly defined.** | **7** | **7** | **5** | **6** | **2** | **6** | **7** |
| **3. Rigor of development** | **7. Systematic methods were used to search for evidence.** | **1** | **4** | **3** | **1** | **2** | **1** | **7** |
|  | **8. The criteria for selecting the evidence are clearly described** | **1** | **1** | **1** | **1** | **1** | **1** | **7** |
|  | **9. he strengths and limitations of the body of evidence are clearly described** | **1** | **1** | **1** | **1** | **1** | **1** | **7** |
|  | **10. The methods for formulating the recommendations are clearly described.** | **1** | **3** | **1** | **1** | **1** | **1** | **7** |
|  | **11. The health benefits, side effects, and risks have been considered in formulating the recommendations** | **1** | **3** | **1** | **3** | **1** | **3** | **5** |
|  | **12. There is an explicit link between the recommendations and the supporting evidence.** | **1** | **1** | **1** | **1** | **1** | **2** | **7** |
|  | **13. The guideline has been externally reviewed by experts prior to its publication.** | **2** | **2** | **1** | **2** | **1** | **1** | **7** |
|  | **14. A procedure for updating the guideline is provided.** | **1** | **3** | **1** | **1** | **1** | **1** | **7** |
| **4. Clarity of presentation** | **15. The recommendations are specific and unambiguous.** | **6** | **6** | **6** | **7** | **6** | **5** | **7** |
|  | **16. The different options for management of the condition or health issue are clearly presented.** | **6** | **6** | **6** | **7** | **5** | **5** | **7** |
|  | **17. Key recommendations are easily identifiable.** | **5** | **5** | **4** | **5** | **6** | **5** | **5** |
| **5.Applicability** | **18. The guideline describes facilitators and barriers to its application.** | **1** | **3** | **3** | **4** | **2** | **3** | **7** |
|  | **19. The guideline provides advice and/or tools on how the recommendations can be put into practice.** | **1** | **1** | **1** | **2** | **1** | **3** | **5** |
|  | **20. The potential resource implications of applying the recommendations have been considered.** | **2** | **4** | **2** | **4** | **3** | **5** | **7** |
|  | **21. The guideline presents monitoring and/or auditing criteria.** | **1** | **2** | **1** | **1** | **1** | **1** | **6** |
| **6. Editorial independence** | **22. The views of the funding body have not influenced the content of the guideline.** | **5** | **5** | **5** | **6** | **4** | **4** | **6** |
|  | **23. Competing interests of guideline development group members have been recorded and addressed.** | **1** | **1** | **1** | **4** | **4** | **4** | **6** |
